# Editorial Chit Chat

**Published:** 1870-07

**Authors:** 


### EDITORIAL CHIT CHAT.

M. NELATON, the celebrated French Surgeon, is suffering from some "heart disease," and has withdrawn from practice.

SPOTTED FEVER.—One hundred and fifteen persons died in Coffee county, Ga., from this disease, in the month of March.

THE SICK.—The *Medical Record* says there are 1,360,000 "constantly sick" persons, or twenty-four to each physician, in the United States.

LADY PHYSICIAN.—Miss Morgan, an English lady, has been graduated with distinction in medicine, surgery and midwifery, at the University of Zurich.

A NEW ALLOY.—A new alloy, composed of hydrogen and mercury, called hydrogenium amalgam, has been discovered by Prof. Oscar Loew, of New York.

FOOD REQUIRED.—A man requires 1.23 of his weight in daily food in order to supply the waste of tissue continually going on in his body; if the waste becomes greater than the repair, death must ultimately ensue.

PERSONAL.—Mr. B. H. Pratt, the accomplished scholar and writer, has charge of the Academy in Danville, Pa. The school is a large and flourishing one—it could not be otherwise under such a management.

IT DON'T PAY.—Dr. Schlesinger, of Berlin, states that the average income of the physicians of that city, is about 600 thalers—$450—per year. Why, that wouldn't more than pay the rent of a respectable office in this city.

DELICATE.—It has been demonstrated that a millionth part of a grain of quinine can be detected by means of the Polariscope. The nicest means of chemistry fails to recognize so small an atom. Verily, we progress in science.

A TRANCE.—We see a case noted in the *London Medical Mirror.* The wife of a blacksmith who had been a short time ill, was supposed to have died, and was placed in her coffin ready for burial. As the lid was being screwed down, the body was noticed to move. She made a very narrow escape from being buried alive.

GOUT.—One of the most comprehensible descriptions of gout we have ever heard, is that one given by a celebrated French physician.—He says: "Place your joint in a vice and screw it up until you can bear it no longer. That may represent rheumatism. Then give the instrument another twist, and you have the gout."

---

THE FIRST SURGEONS.—Æsculapius and his two sons, Muchaon and Podalirius, are the first recorded Greek surgeons. The father flourished about fifty years before the Trojan war.

Hippocrates performed the operation of lithotomy in his day. It is one of the most difficult known to surgeons.

---

A NEW THEORY!—An Oxford professor states that the vast forests of sea weeds, which grow at the bottom of the ocean, produce petroleum! He says the heat from the interior of the earth under the bottom of the sea, distils the sea weed and liberates the oxygen from the hydrogen of the surrounding water and forms petroleum.

---

THE FOURTH is to be celebrated in fine style, in this city. This is the great day for the juveniles. We do not know which day is most prized by them, the "fourth" or Christmas.—Give the children lots of "crackers" and let them fire away—they enjoy it and all should rejoice in their merriment.

---

THE CHOLERA.—Precautions are now being taken to prevent the spread of cholera, abroad. The Sultan of Turkey has sent a commission to found a hospital near the straits of Bab-el-mandeb, to be conducted by a noted French physician. The cholera has been raging for three months in Persia, and ships from Persian ports undergo ten days quarantine.

---

IT CAN'T BE DONE.—We have tried superintending the building of a house—attending the inmates of an hospital and an Eye and Ear Institue—have endeavored to entertain a house full of company, and edit a medical journal, all in the same month, and are thoroughly satisfied that it cannot be successfully accomplished.

---

CHINESE MEDICINES.—Our Chinese friends have a curious system of medicine. It has, to recommend it, its wonderful simplicity. Their prescriptions are divided into seven categorys, viz.: 1st, the Great Prescription; 2d, the Little Prescription; 3d, the Slow Prescription; 4th, the Prompt Prescription; 5th, the Old Prescription; 6th, the Even Prescription; 7th, the Double Prescription. "You pays your money and takes your choice."

---

HARD AND SOFT WATER.—Dr. Letherly, a celebrated English physician, says that moderately hard water is better for drinking purposes than very soft. He also says that a larger precentage of French conscripts are rejected from soft water districts than from where hard water is in use, and that English towns supplied with very hard water have a mortality of four per one thousand less than those whose inhabitants use soft water.

---

LARGE MUSEUM.—The Philadelphians are in exticies over the magnificient museum being erected there for their edification and amusement. The Museum is being built by MESSRS. CARNCROSS & DIXEY, on the corner of 9th and Arch Streets, and when completed and stocked with the animals and curiosities now in waiting for the building, will cost five hundred thousand dollars.

---

ORNITHOLOGICAL.—Our friend Dr. W. H. Gregg, is busily engaged on a new work on ornithology. The Doctor has devoted much time to the study of birds, and has made a very fine collection of the different species which inhabit the United States. He is an excellent Taxidermist as well as Ornithologist and is in every way competent for the task he has undertaken. We are sure his Book will become a standard work on ornithology.

---

THOSE BLANKS.—In our last issue we inclosed a blank and envelope to every one who has been in the habit or receiving the BISTOURY, and who have never paid anything towards its support. Five thousand of those blanks were issued. About one-half have been returned to us with the necessary remittance. We hope to receive many more when this number reaches its destination. To those who have neglected to remit, let this be a reminder that they should do so at once. Remember we are giving you a first class magazine at a very small cost. It is by all odds the cheapest publication in this country. If you do not want the BISTOURY, pay up and order us to stop it—we can easily find a paying customer for it.

A NEW HORROR.—Only think of it Ladies! Those chignons, made of Hindoo bark, and worn by you, are inhabited by little insects called borers, these little rascals creep under the skin of the neck and produce a disease resembling ringworm, though it is much more painful and offensive. We have seen two cases of this disease in this city, we have no doubt that many are troubled with "ringworms" of the neck, that can trace the origin of the disease to the wearing of Hindoo bark upon the head.

---

KNIGHTS TEMPLAR PARADE.—One of the finest parades we have ever witnessed occurred at Williamsport, Pa., on the 16th of June last, upon the occasion of the 17th annual conclave of the Grand Commandery of Knights Templar of the State of Pennsylvania. Over thirty Commanderies were in line, making most a beautiful appearance in their neat uniforms, and with their handsome banners. St. Omer's Commandery, of this city, were present, and went through many evolutions during the time of march, attracting much attention and favorable comment for their superiority in drill.

---

A MEDICAL SHARK.—Some friend has sent us a copy of a little sheet called the "*Medical Star*," and published by one "D. H. Sweeney, M. D.," at New Bloomfield, Pa. We are unable to determine how much of the *Star* is original with its editor, as we find its leading article, headed "American Quackery," copied almost entire from the BISTOURY. Other articles claimed as original in the *Star*, are taken from our journal and mutilated so as to fit the calibre of the *Star* Editor. Doubtless other medical writers furnish the balance of matter displayed in its columns.

---

TEMPORARY INSANITY.—It is said that a large number of ladies residing in New York, are taking steps to cause McFarland to be sent to a Lunatic Asylum. And why not? By what right do our courts of justice judge a man insane and then turn him loose upon the streets to murder the first man who lends a helping hand to his suffering wife? Is his insanity of such a nature that he would not be likely to have a *relapse* under a like train of circumstances?—Perhaps he may find a lunatic to marry him. What protection has a gentleman who may offer assistance to her the first time he feels like kicking her into the street? Shut the lunatic up, or hang him!

THE FIRST DISSECTION.—A friend, now in Venice, writes us that he recently visited the old University of Bologna. This grand old institution once numbered 4,000 students and more than 300 professors, and was the most celebrated medical school in the world. It was here the first human body was dissected and the very first anitomical preparations made. The museum attached to the College is the largest and most complete in the world, both in human and comparative anatomy. Many of the most important chairs were filled, at different times, by women. The University now contains about 500 students, a great falling off since the height of its popularity.

---

PROVOKING.—To have a BISTOURY returned marked "please discontinue," without name or post office attached, or any mark to indicate who the infernal dunce is who returns it. We have received a dozen such since our last issue. We cannot discontinue of course, because we do not know from whom they come. Others return their BISTOURY with their name written upon it, but no post office—the confounded fools think perhaps, that we will look over 20,000 names to find one, little, insignificant address. If you want your journal discontinued pay up like a man, and give your name and address in full. Otherwise the publisher will send it until you do so.

---

TROUTING.—The trout have been unusually plenty on Loyal Sock and Lycoming Creeks, this season. Many large ones have been captured and have furnished rare sport, for the "Sir Isaacs" of this city. We know of no sensation half so pleasant as a pound trout, in a fine pool, attached to the end of a line, (on the "great dun,") and fastened to a seven ounce bamboo-pole, and that same pole in your right hand, while the spotted rascal ("speckled beauty" you've heard before, somewhere,) is jumping and thrashing about in a vain endeavor to free himself. O, isn't it fun to hear the buzz of the reel and the shout of your fat friend on shore, crying "give him more line, Doc!"—"Look out for that root!" while you halloo at every jump he gives, until the farmers in the adjacent fields drop their hoes and run to see the sport? Whew! fun is no name for it--it's glorious—it's indiscribable. Just ask Hamlin or Sanders, they will tell you that it is an experience altogether profitable to have. We can't do the subject justice.

THAT NEW BEVERAGE.—Since the evangelical and spiritual scandal in New York, in gratification of a laudable curiosity to know things, we have yielded to an invitation on the part of an imitative publican, and have tasted gin and milk—*tasted* mind you, and tasted only, for, as the fellow said of the boiled crow, that, notwithstanding we can "worry down" gin and milk, we can't say that we "hanker after it." If g. and m. were the only temptation in the tipple line liable to come in our way, the chances might in that event, be considered favorable that we would die a sober man. We are confirmed in first impressions of the beverage, and agree with the New York *World* that the combination of two such elements as, gin and milk is inartistic, and grates harshly upon every nerve of genteel indulgence. But what could be expected of one of the Smith family who spells his name with a "y?" But the mischief has been done, and at this present moment half the neophyte inebriates in this city are probably fuddling their weak brains with gin and milk—bah!

CHURCH DRESS.—We verily believe that most ladies would rather dress "within an inch of their lives," for church attendance, and render themselves miserably uncomfortable during the service, in order to make a fine appearance in the presence of their neighbors, than to dress neatly and comfortably and brave the danger of church critisism. At Mr. Beecher's last lecture at the Opera House, we observed a couple of ladies dressed in deep mourning, and almost smothered in large black veils, which completely encased their heads and faces; one of them plied her broken fan with so much vim and determination to keep cool, that she disturbed everybody in her neighborhood, with its abominable clatter. That woman was uncomfortable—nearly suffocated in fact; yet that outragious black veil was a necessary part of her make-up—she wouldn't make a proper appearance without it; hence, fumed, sputtered and fanned she.

We believe there is such a thing as mourning for the loss of dear ones, without advertising the fact to the public. We don't think it is necessary to smother ones self in abominable black, at church, or anywhere else, and call it *mourning*. The practice is as unhealthy as it is uncomfortable.

EH?—An exchange speaks of "a man with one eye named Mike O'Cool," but forgets to add the name of his other eye. A strange over-sight.

KILLING FISH WITH GIANT POWDER.—A new and most outrageous fashion of killing fish with "giant" blasting powder has lately been introduced at Carson and other places in Nevada, and this is the way it is practiced: They take a cartridge of giant powder, weighing about a quarter of a pound, insert into it a piece of fuse, properly capped, about six inches in length, then lighting the fuse, the cartridge is thrown into any deep hole supposed to contain trout or other fish. After the cartridge has been thrown into the water, smoke and bubbles of gas are seen to rise to the surface, then in a few seconds comes the explosion—a dull, heavy report. The surface of the water is seen to bulge up, and the ground can be felt to shake for fifteen or twenty feet back from the water. Immediately after the explosion all the fish that happen to be within a circle of twenty-five or thirty feet of the spot where the cartridge fell, come to the surface, either killed outright or so badly stunned that it is some minutes before they recover. With two cartridges over fifty pounds of fish were killed, counting trout, whitefish and chubs. In places after a blast the whole surface of the water would be covered with minnows, from an inch to three or four inches in length. At Elko they are practicing the same style of fishing, only that out there they tie the cartridge to the end of a long pole and thrust it into the water, holding it till the explosion occurs. This is the most destructive mode of fishing ever heard of—it is wholesale slaughter of great and small, good and bad. Should the practice gain ground, it will be necessary for the Legislature to put a stop to it by an act making it a criminal offense to fish with giant powder. Parties have already been trying this blasting process in Lake Tahoe, where by using large cartridges they expect to bring up hundreds of trout at a single shot.

THE FORCE OF IMAGINATION.—Buckland, the distinguished geologist, one day gave a dinner, after dissecting a Mississippi alligator, having asked a good many of the most distinguished of his classes to dine with him. His house and all his establishment were in good style and taste. His guests congregated. The dinner-table looked splendidly with glass china and plate, and the meal commenced with excellent soup. "How do you like the soup?" asked the doctor, after having finished his own plate, addressing a famous gourmand of the day. "Very good, indeed," answered the other; "turtle, is it not?

I only ask because I do not find any green fat." The doctor shook his head. "I think it has somewhat of a musky taste," says another; "not unpleasant but peculiar." "All alligators have," replied Buckland, "the cayman peculiarly so. The fellow I dissected this morning, and which you have just been eating——" There was a general rout of guests; every one turned pale. Half a dozen started from the table; two or three ran out of the room, and only those who had stout stomachs, remained to the close of an excellent entertainment. "See what imagination is," said Buckland. "If I had told them it was turtle, or terrapin, or bird's nest soup,' salt water amphibia, or flesh or the gluten or a fish from the maw of a sea-bird, they would have pronounced it excellent, and their digestion would have been none the worse. Such is prejudice." 'But was it really an alligator?" asked a lady. "As good a calf's head as ever wore a coronet," answered Buckland.

A NEW KIND OF LAMP.—At a Meeting of the British Association for the Advancement of Science, not long ago, a lamp was exhibited which transmits light through living bodies.—Small animals become as if transparent; and the thinner parts of the human body, especially in a child, are quite disaphanous. We have heard before of "letting daylight shine through a man," but it was not done in precisely this way.

